# Organizational culture and leadership behaviors: is manager’s psychological health the missing piece?

**DOI:** 10.3389/fpsyg.2023.1237775

**Published:** 2023-09-28

**Authors:** Julie Dextras-Gauthier, Marie-Hélène Gilbert, Justine Dima, Laetitia Bomoya Adou

**Affiliations:** ^1^Management Department, Faculty of Business Administration, Laval University, Quebec, QC, Canada; ^2^School of Management and Engineering Vaud, Yverdon-les-Bains, Switzerland

**Keywords:** organizational culture, leadership, psychological health, well-being, psychological distress

## Abstract

**Background:**

In a context where organizations struggle to attract and retain highly qualified workers, organizations need to prioritize the psychological health of employees as a retention factor. To do so, they need to provide a healthy work environment. As an integral part of the employee experience, managers are an important factor in employee retention. In past studies, researchers have focused on the importance of leadership in boosting employees’ health without, however, considering factors encouraging such behavior in managers. Recently, some scholars have become interested in managers’ health as a resource allowing them to adopt good leadership behavior. Indeed, these studies reveal interesting links between managers’ emotional state and their behavior as leaders. Other studies, underscore the importance of considering the organizational context to better understand managers’ psychological health that may influence their leadership behaviors. This study proposes to examine the complex process by which organizational culture influences managers’ psychological health, which acts as a resource favoring the adoption of good leadership behaviors that are known to be constructive and have positive effects on employee.

**Methods:**

Path analyses with the CALIS procedure SAS software, version 9.4 were conducted on a sample of 522 managers in three healthcare facilities in the province of Quebec, Canada.

**Results:**

The results revealed that group culture is associated with the two indicators of managers’ psychological health at work. The results also demonstrated that managers’ psychological distress at work is positively related to transactional and *laissez-faire* leadership styles whereas psychological well-being at work is positively related to transformational and transactional leadership. Concerning indirect associations, there is a significant and positive indirect association between group culture and transformational leadership and there is also a significant and negative association between group culture and *laissez-faire* leadership. Finally, there is also an indirect association between hierarchical culture and transactional leadership.

**Conclusion:**

Our study provides a more in-depth understanding of the relationship between organizational culture and leadership styles. More specifically, our findings highlight the benefits of implementing a group organizational culture to enhance psychological well-being, reduce psychological distress symptoms and promote good leadership behaviors.

## Introduction

In a highly complex and changing environment, organizations has no choice but to keep their employees both healthy and high-performing (Salas-Vallina et al., 2021). This challenge is even more important in a context where organizations struggle to attract and retain highly qualified workers in the current labor shortages. In Canada, the labor shortage is such that organizations are finding it increasingly difficult to attract and retain employees (Statistics Canada, 2022). This situation has jeopardized their growth and the quality of the services they offer, forcing them to delay or even refuse orders and, in some cases, reduce their opening hours (Business Development Bank of Canada, 2021). Faced with this situation, human resources specialists have no choice but to prioritize the psychological health of employees as a retention factor (Di Meglio, 2021). Therefore, providing a healthy work environment becomes an important lever to stand out as an employer of choice (Knies et al., 2022). As an integral part of the employee experience, which refers to the overall, real-life experience an employee has at work (Morgan, 2017; Plaskoff, 2017; Batat Wided, 2022), managers are an important retention factor (Dussault and St-Jacques, 2019). Representing a concrete and visible link between employees and senior management, front line managers are standard bearers of the organizational values and mission. Indeed, through their leadership practices, managers can, among other things, protect or expose their employees to the psychosocial risks of the work environment (e.g., psychological demands, workload) (Chénard et al., 2018; St-Hilaire et al., 2018) while contributing to, or hindering, the establishment of a psychologically safe work climate (Parent-Lamarche and Biron, 2022). In past studies, researchers have focused on the importance of leadership in boosting employees’ health (e.g., Kelloway and Barling, 2010) without, however, considering factors encouraging such behaviors in managers (Tafvelin et al., 2019). Recently, some scholars have become interested in managers’ health as a resource allowing them to adopt good leadership behaviors (e.g., Kaluza et al., 2020). Indeed, these studies reveal interesting links between managers’ emotional state and their behaviors as leaders. Some authors also underscore the importance of considering the organizational context to better understand managers’ psychological health that may influence their leadership behaviors (St-Hilaire and Gilbert, 2019). While organizational culture has already been identified as an important organizational factor in the study of workers’ health (e.g., Dextras-Gauthier and Marchand, 2016), what about managers’ health? This study proposes to examine the complex process by which organizational culture influences managers’ psychological health, which acts as a resource favoring the adoption of good leadership behaviors that are known to be constructive and have positive effects on employees (Wang et al., 2019). An integrative model will be proposed to put forward contextual analysis of psychological health at work. This is significant considering that critics (e.g., Guest, 2017) have exhorted researchers to go beyond managers themselves and their working conditions to explore how organizational contextual elements influence psychological health at work. Given that the resources necessary to favor managers’ health and well-being are rooted in an organizational context particular to each organization, it appears essential to examine more specifically this context favoring these resources (Chadwick et al., 2015). In doing so, this present study makes four major contributions to the field of psychological health. First, this study contributes to a better understanding of managers’ psychological health who are often forgotten in the literature (Barling and Cloutier, 2017). Second, this study contributes to the literature by considering the organizational context, i.e., organizational culture, as an antecedent to the psychological health of managers. Third, this study allows us to better comprehend how psychological health acts as a resource for managers to the adoption of good leadership behaviors. Finally, this study focuses on managers’ own perspectives, which is particularly interesting in addition to being less studied (e.g., Tóth-Király et al., 2023).

### Organizational culture and manager’s psychological health

Psychological health referred to a multidimensional construct comprising positive (e.g., well-being) and negative (psychological distress, burnout) aspects (e.g., Masse et al., 1998; Keyes, 2003; Westerhof and Keyes, 2010; Gilbert et al., 2011). Although a number of studies emphasize the importance of organizational context in examining psychological health at work (e.g., Chowdhury and Endres, 2010), only a few have actually incorporated organizational culture into their analyses (e.g., Dextras-Gauthier and Marchand, 2016). Organizational culture is a critical component related to the social context of an organization, a factor which has proven to be an important predictor in the process leading to individuals’ well-being (Dextras-Gauthier and Marchand, 2016; Haines et al., 2018). Viewed as a multi-levels concept, Schneider et al. (2013) define organizational culture as the shared values and beliefs characterizing an organization. Most of empirical research in the organizational culture literature takes upon the functionalist view. For the functionalist view, organizational culture has a strong impact on key organizational and individual outcomes such as performance, innovation, and psychological health (Boyce et al., 2015; Dextras-Gauthier and Marchand, 2016), and leadership and organizational culture taken together exert an influence on organizational phenomena (Hartnell et al., 2019; Xenikou, 2022). Organizational culture has been studied as a contextual factor that sets constraints and boundary conditions for organizational phenomena to occur (Erdogan et al., 2006; Xenikou, 2022). Since organizational culture provides the context for the establishment and adoption of management practices, it is in a position to influence not only the organizational structure (Buenger et al., 1996) and working conditions (Dextras-Gauthier et al., 2012), but also the resources leading to manager’s well-being. According to Nielsen et al. (2017), these resources can be found at four different levels: the organization, the leader, the group and the individual himself. Linking the organizational culture with an organization’s available resources to prevent psychological health problems is consistent with the earlier theoretical conceptualization of Peterson and Wilson (2002) suggesting that managerial systems, organizational structures and leadership behaviors act as mediating variables in the association between organizational culture and employees’ health. Consequently, it is proposed that organizational culture influences managers’ psychological health which, in turn, is considered an important resource for these same managers to adopt positive leadership behaviors. Past literature has shown that certain types of organizational culture are beneficial for employees’ well-being (Marchand et al., 2013; Olynick and Li, 2020; Lu et al., 2022). Recently, Lu et al. (2022) found that a patient safety-oriented organizational culture decreases burnout. According to these authors, a patient safety-oriented culture is an organizational resource for employees’ well-being. Moreover, Olynick and Li (2020) found that employees who work in a human-oriented and collaborative culture (group culture) reported lowest levels of stress, followed by those in an innovation/developmental oriented culture and those in a bureaucratic/hierarchical culture. Stress level was higher among workers from an organization with a competitive/rational culture (Olynick and Li, 2020). In addition, Marchand et al. (2013) found that group culture, oriented toward workers and collaboration reduces psychological distress, depression and emotional exhaustion, while increasing well-being. They also found that a more competitive culture has deleterious effects on psychological distress, depression and emotional exhaustion. These authors concluded that group culture is beneficial for employees’ psychological health, while a competitive culture is detrimental.

These studies relied mainly on the Competing Value Framework (CVF) (Quinn and Rohrbaugh, 1983), in which values are central to the organizational culture (Marques et al., 2021). Values are defined as important standards that guide the choice of actions (Van der Wal et al., 2008; Van der Wal and Huberts, 2008; Weske et al., 2020). For Schein (2010), organizational culture has three layers: artifacts which are the most visible elements of culture; espoused values which are less visible than artifacts; and the underlying assumptions that represent the least visible layer of culture. In the CVF of Quinn and Rohrbaugh (1983), culture is derived from competing organizational values.

According to this typology, the culture of an organization is defined along two main axes. The horizontal axis represents whether the organization is focusing on its internal dynamics/characteristics or on the external environment. The vertical axis represents the organization’s orientations in terms of organizational structure, i.e., organizations that focus on stability and control, and those that focus on flexibility and change. Taken together, these two axes define four types of organizational culture: *group, developmental, rational and hierarchical*. The *group culture,* with its emphasis on internal characteristics and dynamics, transposes the idea of flexibility. This type of culture attaches a great importance to the development of its human resources, to autonomy and flexible management processes. *Group culture* emphasizes participation, cooperation, mutual trust, team spirit, individual growth and open communication. This type pf culture fosters commitment and fulfillment at work through the development of human resources (Cameron et al., 2006). The *developmental culture,* with its focus on external environment, also emphasizes flexibility. This type of culture values experimentation, innovation, agility, autonomy, professional mobility and entrepreneurship. This culture is based on the ability to learn, creativity, adaptability and organizational expansion (Cameron et al., 1999). The *hierarchical culture,* with its emphasis on internal characteristics and dynamics, is control-oriented. This type of culture seeks to create stability and continuity through information control, division of labor, efficiency, formal procedures and rigid management processes. *Hierarchical culture* also values order, rules, predictability, control, uniformity and stability (Cameron et al., 1999). *Rational culture* emphasizes control and focuses on the external environment. This type of culture values rational decision-making, individual and group accountability, performance management and the achievement of organizational goals. *Rational culture* also focuses on organizational productivity, competitiveness and profitability (Cameron et al., 1999). Based on the Competing Values Framework, past empirical results suggest that organizational culture influences employee’s psychological health (e.g., Dextras-Gauthier and Marchand, 2016). Even though most of the studies linking organizational culture and psychological health in the workplace focus on workers, it is possible to believe that the same mechanisms are at work when it comes to the psychological health of managers. Based on these empirical findings and the theoretical conceptualization of Peterson and Wilson (2002), we formulate the following general hypothesis:

*Hypothesis 1*: Organisationnel culture influence managers’ psychological health.

### Healthy managers and leadership behaviors

The most studied leadership styles are rooted in the full range leadership model (Burns, 1978; Bass, 1985). Transformational leadership refers to leaders who influence their followers through charisma, inspire them and stimulate them to engage in creative thinking and consider the needs of each follower (Avolio and Bass, 1995). On their part, transactional leaders establish reward for followers who perform in their job assignments, they are more focused on the tasks and process of sharing information and resources so that employees are able to get the job done (Judge and Piccolo, 2004). The last leadership style is the *laissez-faire* leadership which refers to leaders who are absent, avoid making decisions, and hesitate to take any kind of actions (Judge and Piccolo, 2004). In the literature, studies highlight the benefits of positive leadership practices for employees’ functioning (e.g., Harms et al., 2017; Montano et al., 2017; Gill et al., 2018; Broetje et al., 2020; Montano et al., 2023) but few studies have considered the antecedents of leadership (Avolio et al., 2009; Nielsen and Cleal, 2011; Turgut et al., 2020). Tafvelin et al. (2019) recently claimed that “leading well is a matter of resources,” stressing the importance of leaders’ well-being and working environment for their capacity to adopt good leadership behaviors. However, limited attention has been paid to the boundary conditions that enable managers to deploy positive leadership practices (Nielsen and Taris, 2019). These boundary conditions relate to the conditions experience by managers such as their own health and well-being (Nielsen and Taris, 2019). These conditions may influence a manager’s ability to adopt good leadership behaviors (Nielsen and Taris, 2019; Tafvelin et al., 2019). Moreover, in line with Hobfoll (1989) as well as Halbesleben et al. (2014), the assumed antecedent of leadership behaviors can be categorized as resources (Turgut et al., 2020). Hobfoll (1989) defined resources as those “objects, personal characteristics, conditions, or energies that are valued by the individual or that serve as a means for attainment of these objects, personal characteristics, conditions, or energies” (p. 516). Based on this definition, Halbesleben et al. (2014) redefined resources more specifically as “anything perceived by the individual to help attain his or her goals” (p. 1,338). Resources can be divided into multiple levels of the organization: individual, group, leader, organization and overreaching context (Nielsen et al., 2017). Recently, Tafvelin et al. (2023) underscore the importance of leaders’ roles clarity and reasonable workload to prevent passive destructive leadership (e.g., avoidance). They also show the link between leaders’ stress and active destructive leadership (e.g., threats). Also, Tóth-Király et al. (2023) highlighted the importance of job control and recognition in addition to a low level of workload to predict higher levels of good leadership behaviors which are associated with burnout. Resources may be inherent within the individual, so, managers’ own well-being could be considered an important resource for them. According to the Conservation of Resources (COR) model (Hobfoll, 1989), individuals strive toward retaining or increasing their resources. However, little is known about the relationship between managers’ well-being and their leadership behaviors (Kaluza et al., 2020).

As noted by Barling and Cloutier (2017), while employees’ health has been studied many times, managers’ health has remained under the radar. It is only in the last few years that researchers have become more interested in managers’ psychological health (e.g., St-Hilaire and Gilbert, 2019). Indeed, the literature reveals a positive relation between leaders’ depressive and anxiety symptoms (Byrne et al., 2013), burnout (e.g., Arnold et al., 2015, 2017), and stress (Harms et al., 2017) and their negative/abusive leadership. In their study, Nielsen and Taris (2019) found that anxious managers exerted lower levels of transformational leadership and high levels of *laissez-faire* leadership over time. Tafvelin et al. (2019), meanwhile, argued that managers who already are in possession of resources (as such their own well-being) may use these resources to mobilize transformational leadership behaviors. These empirical findings show that in order to unleash the benefits that good leadership behaviors can have for followers’ psychological health, more focus need to be put on managers’ context and resources such as their own psychological health. This is even more important since in recent years, the health and working conditions of managers have deteriorated (Dellve et al., 2017; Dellve and Eriksson, 2017; Gilbert et al., 2019). For example, the study of Fiedler et al. (2018) reveals that 10.3% of managers’ report depressive symptoms and that 25.4% are considered to have a low level of well-being. Moreover, several studies reported stressful working conditions and high turnover among managers specifically in sector like healthcare system (e.g., Gilbert et al., 2019; Corbière et al., 2020). These stressful working conditions include, for example, conflict of values and loyalties, high-performance pressure, hectic work pace and long working hours (Larsson et al., 2015; Tengblad, 2017; Gilbert et al., 2019). Since managers operate in a specific context, and the conditions under which they lead (including their own inherent resource such as their psychological health) will influence their capacity to adopt good leadership behaviors (Nielsen and Taris, 2019), we thus considered their well-being as an important resource which encourage the adoption of positive leadership behaviors (Kaluza et al., 2020). Given that managers, particularly through their leadership and management practices, play a central role with their team in terms of psychological health and also on their individual performance (Sonnentag, 2015; Inceoglu et al., 2018; St-Hilaire and Gilbert, 2019), more knowledge is necessary to better understand the relationship between managers’ psychological health and their leadership behaviors (Kaluza et al., 2020). A better comprehension of this relation could not only benefit managers themselves but also their team members and the entire organization. Consequently, we formulate the following general hypotheses:

*Hypothesis 2*: Managers’ psychological health influence their leadership behaviors.

*Hypothesis 3*: Organizational culture influence leadership behaviors through the mediation effect of psychological health of managers.

**Figure 1:**
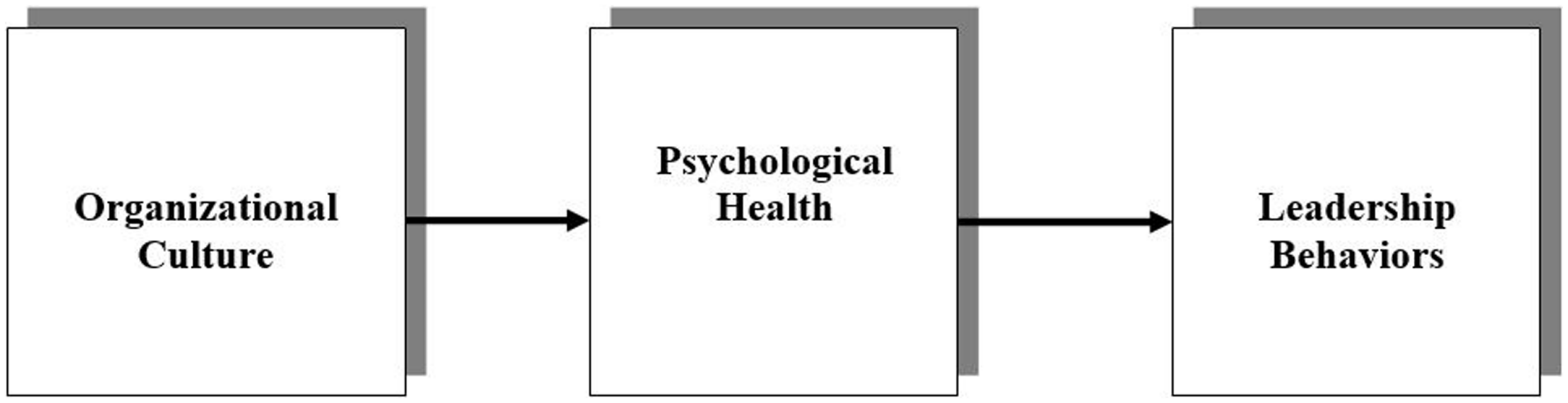
An integrative model of the influence of organizational culture on leadership behaviors through manager’s psychological health.

The conceptual model developed to test our hypotheses is illustrated in Figure 1.

## Materials and methods

### Participants and procedure

The data used in this paper come from two separate studies, both driven by the overarching research objective of better understanding leadership behaviors and the psychological health of Quebec healthcare managers. The combination of these data sets was necessitated by the need to have a sufficient number of participants, essential for conducting the requisite statistical analyses to address the research questions. As Zickar and Keith (2023) noted, in many cases, it is a challenge for researchers to get enough participants to complete questionnaires. Given that both of these studies shared a common research objective, a same population with similar characteristics (managers in a Quebec healthcare facility), a same sector of activity (healthcare sector) with organizations driven by the mission of providing public health services, the inclusion of the data from these two studies not only effectively addressed our research questions but also upheld the integrity of data quality, as emphasized by Zickar and Keith (2023). Moreover, the questionnaires were also distributed at roughly the same point in time, spanning the summer and autumn of 2019, before the onset of the pandemic. In both studies, the participants answered a secure online questionnaire *via* Lime Survey software between July 2019 and September 2019 in the first study (n_1_ = 78; response rate: 83%) and between July 2019 and December 2019 in the second study made of two organizations (n_2_ = 294; response rate: 78% n_3_ = 150; response rate: 25%). The questionnaire addressed several themes including organizational culture, psychological health at work, leadership behaviors and socio-demographic characteristics. The electronic questionnaire was sent through email to managers from three healthcare organization in Quebec. The survey took approximatively 30 min to be complete. The total sample consists of 522 managers (68.3% woman, M_age_ = 45.23, SD_age_ = 7.89). Among participants, 47.89% were frontline managers, 32.57% were intermediate managers and 19.54% were executive managers. Participants had an average tenure of 13.38 years (SD = 3.43 years) in their organization. They also had an average tenure of 8.96 years (SD = 2.9 years) in a management position.

### Measures

#### Organizational culture

Organizational culture was measured, in both studies, with the Organizational Culture Profile (OCP), a scale measuring organizational values developed by O'reilly et al. (1991). Participants completed the French version of the OCP validated by Evraert and Prat dit Hauret (2003). The scale contains 26 items that correspond to values likely to match the culture of an organization (e.g., fairness, stability, predictability, tolerance, competitiveness). Participants were to indicate to what extent the various values described the culture of their organization on a 5-point Likert-type scale ranging from *not at all (1)* to *a great extent (5)*. Following the recommendations of Marchand et al. (2013), three items were removed from the scale for the analyses. As so group culture includes 7 items (alpha = 0.85), hierarchical culture 6 items (alpha = 0.69), developmental culture 5 items (alpha = 0.81), and rational culture 5 items (alpha = 0.79). The results of Marchand et al. (2013) also support the use of the OCP scale for measuring organizational culture in a way that is consistent with the Competing Value Framework. Examples of items for the group culture are: *fairness, respect for the individual’s rights, tolerance.* Examples of items for the hierarchical culture are: *being rule oriented, security of employment, stability.* Examples of items for the developmental culture are: *a willingness to experiment, not being constrained by many rules, being innovative.* Examples of items for the rational culture are: *being competitive, being results oriented, having high expectations for performance.*

#### Psychological health at work

In both studies, managers completed the short, validated version (Gilbert and Malo, 2017) of the psychological health at work questionnaire of Gilbert et al. (2011), based on the work of Masse et al. (1998). This questionnaire focuses on the subjective experience (affects and cognitions) of workers in the specific context of work. It measures psychological well-being at work (positive aspect) and psychological distress at work (negative aspect). This questionnaire includes a total of 21 items, 12 items for the psychological distress at work (alpha = 0.87) and 9 items for the psychological well-being at work (alpha = 0.88). Examples of items of the psychological well-being at work are: *Lately, in my job, I feel emotionally balanced, I feel appreciated by others, I have goals*. For the psychological distress at work, examples of items are: *Lately, in my job, I lack confidence in myself, I feel arrogant with others, I feel undervalued*.

#### Leadership behaviors

Based on a categorization widely used in the leadership literature and advocated by several authors (e.g., Avolio and Gardner, 2005; Schyns and Schilling, 2013), we divide leaderships behaviors in two broad categories: positive and negative leadership behaviors. To measure positive leadership behaviors, we used transformational and transactional leadership (Wang et al., 2019). To measure negative leadership behaviors, we used the *laissez-faire* leadership (Judge and Piccolo, 2004). The first study used the short version of the multifactor leadership questionnaire (MLQ-5x) (Bass and Avolio, 1989) validated in French by Cacciatore (2015) and adapted to be self-reported. The instrument includes a total of 36 items. For our analyses we have retained only 32 items: 20 items for the transformational leadership (α =0.84), 8 items for the transactional leadership (α =0.58) and 4 items for the *laissez-faire* leadership (α =0.49). Because in transactional leadership, we find both active and passive types of leadership, and passive leadership overlap with *laissez-faire* leadership, we only considered the items on active leadership behaviors (contingent rewards and management by exception-active) (Judge and Piccolo, 2004; Montano et al., 2023). An example of item for the transformational leadership is *I speak optimistically about the future*, for transactional leadership *I express my satisfaction when my expectations are met*, and for leadership *laissez-faire I am not available*.

In the second study, we used the Leadership Self-Report Scale of Dussault et al. (2013) composed of 21 items. This questionnaire is also based on the Multifactor Leadership Questionnaire (MLQ) of Bass and Avolio (1989) and measure the same three dimensions of transformational leadership (12 items, α = 0.78), transactional leadership (6 items, α = 0.78) and *laissez-faire* leadership (3 items, α = 0.56). We used a Likert scale to measure items range from 1 (completely disagree) to 4 (completely agree). An example of item for transformational leadership is *I communicate my vision of the future*, for transactional leadership *I recognize when staff do good work*, and for leadership *laissez-faire I am not available*.

It is worth mentioning that the two leadership measurement tools used in this study were both based on the work of Bass and Avolio (1989). However, given that this is two different measures, leadership data have been standardized to bring it back on the same scale. Regarding the mechanism used to standardize the leadership data, the averages for the three types of leadership in the first study, which ranged from 0 to 4, were multiplied by 0.75 and then added by 1 to obtain scores between 1 and 4, as in the second study. Finally, it is also important to note that the Cronbach’s alphas obtained in this study, particularly in relation to *laissez-faire* leadership, are low (below 0.7). However, considering that this type of leadership is challenging to measure (Dussault et al., 2013), it is not uncommon to obtain a lower coefficient (Tóth-Király et al., 2023).

#### Ethical considerations

Both studies are part of a larger joint research project that has obtained the approval of all three healthcare facility’s research ethics committee. To protect the confidentiality of the participants, it was mentioned in the invitation sent to the managers that this research was anonymous and confidential. All of the participants gave their informed consent and no compensation was granted to the participants.

#### Analysis

Following preliminary analyses, we conducted path analyses with the CALIS procedure SAS software, version 9.4. Those analyses focus on understanding the direct and indirect association based on Preacher and Hayes (2004) method. Path analysis, a subcategory of Structural Equation Modeling (SEM), allows researchers to assess the complex interplay and associations between several variables (Barbeau et al., 2019). This statistical technique provides insights to the underlying processes and mechanisms of a given phenomenon. More precisely, Preacher and Hayes (2004) method allowed us to determine whether the association between organizational culture and leadership behaviors is mediated by manager’s psychological health. Our model was tested with the full information maximum likelihood estimation method (FIML estimation). The goodness-of-fit was assessed using the Comparative fit Index (CFI) and the Tucker-Lewis Index (TLI). Values above 0.95 for the CFI and TLI indicate an excellent fit (Hoyle, 1995).

## Results

Table 1 presents the means and standard deviations for all the variables studied. Overall, respondents seem to have a high level of psychological well-being. Among the four types of organizational culture, rational culture stands out as the one most prominently perceived by the respondents. Finally, respondents indicate a greater inclination toward adopting transformational leadership behaviors compared to the other two types of leadership behaviors.

**Table 1:** Descriptive statistics.

	*M*	*SD*
Group Culture	3.83	0.59
Hierarchical Culture	3.71	0.53
Developmental Culture	3.60	0.67
Rational Culture	4.17	0.57
Psychological distress	2.25	0.70
Well-being	5.92	0.65
Transformational leadership	3.60	0.29
Transactional leadership	3.34	0.46
*Laissez-faire* leadership	1.30	0.42

*M*, mean; SD, standard deviation.

The results of our analysis show acceptable fit indices for the tested model (*χ*^2^ *= 29.281; dl = 12; p = 0.004; CFI = 0.986; TLI = 0.0.958; RMSEA = 0.053; SRMR = 0.047*). The CFI and TLI values are greater than 0.95, which indicate a good fit with the data. However, the RMSEA value is greater than 0.05. Several authors recognize, that an RMSEA greater than 0.05 is acceptable, for small sample sizes or those comprising only a small number of degrees of freedom (e.g., Kenny and Judd, 2014; Kenny et al., 2015).

Table 2 presents the direct effects of each type of organizational culture on managers’ psychological health and the direct effects of managers’ psychological health on their leadership behaviors.

**Table 2:** Direct effects of organizational culture on managers’ psychological health and managers’ psychological health on their leadership behaviors.

			Estimate	SE
Group culture	===>	Psychological distress	−0.329**	0.059
Group culture	===>	Well-being	0.251**	0.059
Hierarchical culture	===>	Psychological distress	0.040	0.055
Hierarchical culture	===>	Well-being	0.086	0.054
Developmental culture	===>	Psychological distress	0.001	0.056
Developmental culture	===>	Well-being	0.069	0.055
Rational culture	===>	Psychological distress	0.023	0.050
Rational culture	===>	Well-being	−0.045	0.049
Psychological distress	===>	Transformational leadership	0.081	0.059
Psychological distress	===>	Transactional leadership	0.164*	0.061
Psychological distress	===>	*Laissez-faire* leadership	0.241**	0.059
Well-being	===>	Transformational leadership	0.427**	0.057
Well-being	===>	Transactional leadership	0.257**	0.062
Well-being	===>	*Laissez-faire* leadership	−0.037	0.061

**p ≤ 0.01; *p ≤ 0.05.

Firstly, concerning the direct effects of each type of organizational culture on managers’ psychological health, the results highlight a significant and negative association between group culture and psychological distress. There is also a significant and positive association between group culture and well-being. However, there is no significant effect between the other types of organizational culture (hierarchical, developmental and rational) and managers’ psychological health.

Secondly, concerning the direct effects of managers’ psychological health on their leadership behaviors, the results obtained indicate that managers’ psychological distress is positively associated with transactional and *laissez-faire* types of leadership. There is no significant effect between managers’ psychological distress and transformational leadership. Our results also indicate that managers’ well-being is positively associated with transformational and transactional types of leadership. There is no significant effect between managers’ well-being and *laissez-faire* leadership. Table 3 presents the results of the indirect (mediation) effects of the managers’ psychological health in the relation between organizational culture and their leadership behaviors.

**Table 3:** Indirect effects of organizational culture on managers’ leadership behaviors.

Type of culture		Psychological health		Type of leadership	Estimate	SE
Group culture	===>	Psychological distress and Well-being	===>	Transformational	0.081**	0.026
Group culture	===>	Psychological distress and Well-being	===>	Transactional	0.010	0.019
Group culture	===>	Psychological distress and Well-being	===>	*Laissez-faire*	−0.089**	0.021
Hierarchical culture	===>	Psychological distress and Well-being	===>	Transformational	0.040	0.022
Hierarchical culture	===>	Psychological distress and Well-being	===>	Transactional	0.029*	0.013
Hierarchical culture	===>	Psychological distress and Well-being	===>	*Laissez-faire*	0.007	0.016
Developmental culture	===>	Psychological distress and Well-being	===>	Transformational	0.030	0.021
Developmental culture	===>	Psychological distress and Well-being	===>	Transactional	0.018	0.011
Developmental culture	===>	Psychological distress and Well-being	===>	*Laissez-faire*	−0.002	0.016
Rational culture	===>	Psychological distress and Well-being	===>	Transformational	−0.018	0.016
Rational culture	===>	Psychological distress and Well-being	===>	Transactional	−0.008	0.010
Rational culture	===>	Psychological distress and Well-being	===>	*Laissez-faire*	0.007	0.014

**p ≤ 0.01; *p ≤ 0.05.

Lastly, concerning indirect associations, there is a significant and positive indirect association between group culture and transformational leadership. In other word, the higher the group culture is, the higher the level of transformational leadership. Group culture impacts transformational leadership through managers’ psychological health (well-being: estimate = 0.107; psychological distress: estimate = −0.027). There is also a significant and negative indirect association between group culture and *laissez-faire* leadership. Thus, the higher the group culture is, the lower the laissez faire leadership. Group culture also impacts laissez faire leadership through managers’ psychological health (psychological distress: estimate = −0.080; well-being: estimate = −0.010). Our results also show that there is a significant and positive association between hierarchical culture and transactional leadership. So, the higher the hierarchical culture is, the higher the transactional leadership. Hierarchical culture impacts transactional leadership through managers’ psychological health (well-being: estimate = 0,022; psychological distress: estimate = 0,007). There is no significant indirect association between developmental culture and managers’ leadership behaviors. There is also no significant association between rational culture dans managers’ leadership behaviors.

## Discussion

The study’s main objective was to better understand how organizational culture influence managers’ leadership through the psychological health of managers. To do so, direct and indirect relationships between organizational culture, managers’ psychological health, and managers’ leadership behaviors were investigated. In our study, the broader hypotheses find support as group and hierarchical cultures exerts an influence on leadership behaviors through the psychological health of managers. However, given the specificity of our analyses, the results reveal that only a few paths are statistically significant. Not all types of culture in our sample have a significant impact on leadership behaviors through the psychological health of managers. Consequently, this underscores the importance for a cautious approach to interpretation, emphasizing the need for further research in this domain. Nevertheless, the specificity of our analyses has led us to develop a finer understanding of these complex relationships. Several authors highlight the importance for managers to recognize aspects of the social context and adapt their leadership accordingly (Schein, 2010; Klimoski, 2013; Hartnell et al., 2016). However, prior research has offered limited insight into the potential mediators within the relationship between organizational culture and leadership behaviors (Hartnell et al., 2016, 2019). Our results contribute to addressing this gap by shedding light on potential mediators within this relationship. Indeed, our results show that group culture is associated with the two indicators of managers’ psychological health at work. Such findings support the use of the group culture as a significant factor for studying occupational health at work. Given that the group culture puts a strong emphasis on human resources and promotes a supportive and healthy work environment, managers in work environment characterized by this culture type might experience less work stress that may explain why they seem to report fewer levels of psychological distress and higher levels of well-being. These results are consistent with previous research on organizational culture and employees’ psychological health at work (Marchand et al., 2013; Dextras-Gauthier and Marchand, 2016). Our results also show that there is a significant and positive association between hierarchical culture and transactional leadership. According to Weske et al. (2020), organizational values of public organizations are traditionally associated with bureaucracy and characterized by impartiality, legality and neutrality. However, with the introduction of the New Public Management principles in public organizations more emphasis is put on incentives and rewards for individuals and organizations that achieve their targets (Bach, 2019). Without abandoning their hierarchical/bureaucratic culture and their traditional investment in employees’ well-being (Knies et al., 2022), public organization put forward a performance management system based on incentive structures and targets that are now a defining feature of the public sector context (Bach, 2019). The tighter control put on performance management (Bach, 2019) could explain the association with transactional leadership since transactional leadership represents managers contingent rewards and corrective actions (Bass and Avolio, 1989). Our findings also highlight the relevance of organizational culture in future studies on managers’ psychological health at work. By including organizational culture, and specifically group culture in their study, researchers would have a more complete perspective of the contextual factors that are directly or indirectly associated with psychological health at work. Moreover, organizational culture is not only a context in which a manager lead, organizational culture’s effectiveness depends on manager’s leadership behaviors (Hartnell et al., 2016, 2019) and as our results show, managers’ leadership behaviors are influence by managers’ psychological health.

More specifically, our results demonstrated that managers’ psychological distress at work is positively related to transactional and *laissez-faire* leadership styles whereas psychological well-being at work is positively related to transformational and transactional leadership. Those results are interesting because transactional leadership style is both related with positive and negative aspects of psychological health. Transactional leadership style focused on goal achievement (Judge and Piccolo, 2004). A manager with a high level of psychological well-being can adopt transformational leadership but also transactional leadership given that these two styles are considered complementary and positive (Judge and Piccolo, 2004). The results also suggest that a manager with a high level of psychological distress could use transactional leadership to do the minimum in his role (provide the necessary resources to employees to be able to achieve their objectives) and even use *laissez-faire* leadership. These results are in line with those of Arnold et al. (2017) who showed that managers who adopted a more passive leadership style reported more burnout. Given the current discussion on construct proliferation in leadership research (e.g., Banks et al., 2018; Bormann and Rowold, 2018; Montano et al., 2023), our results show that all three types of leadership are important to considered. Our results are in line with previous research on managers’ psychological health and leadership behaviors with negative leadership behaviors is associated with higher levels of managers’ psychological distress (e.g., Kaluza et al., 2020) and positive leadership behaviors is associated with higher levels of managers’ well-being (Tafvelin et al., 2019). Our results underline the importance of managers’ own psychological health for the effectiveness of leadership behaviors.

### Practical contributions

Our results imply that in order for a manager to adopt good leadership behaviors, organization must put more focus on managers’ context and working conditions. As so, organizations must improve manager’s resources and reduce their work constraints (Gilbert et al., 2019). In recent years, the health and working conditions of the managers themselves have deteriorated especially in the healthcare sector (Dellve and Eriksson, 2017). In this particular sector, several authors point out higher level of stress, and more stressful work conditions among managers (Gilbert et al., 2019; Corbière et al., 2020). Organizations could improve managers’ psychological health through interventions aimed at building workplaces resources at multiple levels in the organization. Following the IGLOO model developed by Nielsen et al. (2017), organizations can improve resources at four level: individual (e.g., self-efficacy, competence, self-esteem), group (e.g., social support between colleagues), leader (e.g., social support from the manager), organization (e.g., autonomy, skills variety, HR practices) and overreaching context, the global context in which an organization is nested (e.g., social context, national context and legislation).These five resources levels echo the categorical framework of Johns (2006) such as the omnibus context (e.g., national culture, institutional forces) and the discrete context (e.g., task characteristics, job characteristics, social network). Looking more specifically at one level of resource, our results show that organizational culture, and in particular group culture, can act as a resource for preserving managers’ psychological health. Moreover, in a sector where organizational changes and crises are frequent and numerous (Lyet and Molina, 2019), as is the case in the healthcare sector, it is important for healthcare establishments to question their organizational culture and the consequences associated with this type of culture. Changing an organizational culture is certainly a challenge. However, it is possible to activate a lasting transformation of culture beyond the surface symbols and rework the values that guide the way work is done. To do so, an organization must first understand its cultural system, i.e., its symbols, myths, rituals, routines, organizational and power structures and control systems. In the case of the healthcare sector, this means questioning, for example, the very strong hierarchical distance, the special status of doctors, the decisive legitimacy given to managers, the hierarchical decision-making structure, the silo structure and the high degree of specialization. Questioning these elements is the first step in changing an organization’s culture and consequently its working conditions. Indeed, recent studies indicated that organizational culture is indirectly associated with psychological health at work *via* the influence it exerts on various element of the working environment and conditions (e.g., psychological and physical demands, rewards, number of working hours, autonomy, skills utilization) (Dextras-Gauthier and Marchand, 2016). Our results show that group culture is positively associated with managers’ well-being and negatively associated with managers’ psychological distress. The main characteristics of group culture is its orientation toward its human resources (Cameron et al., 1999). Thus, group culture is concerned with the well-being and personal development of its employees in the workplace (Cameron et al., 2006). Similarly, group culture seems to reduce those working conditions that are the most toxic for employee and manager’s psychological health (Dextras-Gauthier and Marchand, 2016). For managers in the healthcare sector better working conditions through a group culture could mean, for example, having team that do not exceed 30 subordinates, shared managerial assignments, peer support through colleagues and co-development workshops, reasonable working hours per week and organizational resources such as support from the human resource department (Dellve and Eriksson, 2017; Gilbert et al., 2019). Ensuring adequate working conditions for managers in the healthcare sector is an important factor in a context where the Quebec government is preparing to hire several hundred managers to achieve the objectives set out in Bill 15. This change of attitude toward managers in the healthcare sector comes almost 10 years after the departure of 1,300 managers following a government reform that abolished many middle management positions. The number of executives in the Quebec healthcare sector decreased from 12,115 in 2012 to 9,327 in 2018 [Ministère de la santé et des services sociaux (MSSS), 2019]. These events have had notable impacts on managers who have seen their working conditions deteriorate (Corbière et al., 2020). These contextual factors are important to consider since these institutional constraints describe the context in which leadership takes place (Oc, 2019). Moreover, changes in the omnibus context could shape the discrete context, which in turn could affect leadership behaviors (Oc, 2019). For example, in the healthcare sector, the Ministry of Health guidelines can exert pressure on healthcare establishments that will force them to change or revisit their budget in order to reduce costs. This restrictive environment could affect not only manager’s psychological health at work but also their leadership behaviors. The health of managers in this sector appears to be an important object of study, especially given that these managers operate in a sector of activity that is critical for the well-being of the population and of society in general. Future research could explore different context to enrich our knowledge of mechanisms that translate the effects of contextual factors on manager’s psychological health and leadership. Finally, for organizations, establishing a group culture could be the first step toward creating a more healthy and safe working environment for their managers but also for their employees. This is particularly important since managers’ positive leadership have an influence on followers’ psychological health (Montano et al., 2023). Also, considering the importance placed on leadership development in organizations and all the money invested in leadership training, our results raise the need to challenge the training approach. The development of leadership skills is certainly relevant, but organizations must question the conditions offered to their managers so that they have the necessary resources (e.g., organizational culture and psychological health) to adopt good leadership behaviors. It would be relevant for managers that leadership training includes information about leadership in different organizational cultures and the management of their own psychological health.

### Limits and avenues for future research

This study is not without limitations. First, this is a cross-sectional study which implies that the relationships observed cannot be interpreted causally and will need to be replicated longitudinally. Some inverse relationships are possible because managers with low psychological health could described their organizational culture in negative terms because of their psychological health status. Also, some authors (e.g., Kaluza et al., 2020; Tóth-Király et al., 2023) shown that using good leadership behaviors such as transformational leadership influence managers’ own psychological health in creating a positive and resourceful work environment. Second, it may be that evaluations of organizational culture can also be influenced by a number of individual-related characteristics [e.g., age, gender, nationality, tenure in the organization, education and hierarchical level (Hofstede et al., 1990)]. For example, women may place more importance on specific organizational values, while men may place more importance on others. Moreover, national culture could also influence the values, beliefs and attitudes of individuals (Hofstede et al., 1990), which in turn can have an impact on an individual’s assessment of his organization’s culture. Length of service in an organization can also have an impact on an individual’s perception of his organization’s culture, as the socialization process is a powerful vector for internalizing organizational values (Xenikou, 2022). Further analysis is needed to clarify these possible confounding effects. Third, the data were collected in three healthcare facility located in a Canadian province. Considering that the Quebec health sector has faced multiple upheavals in recent years, our results cannot be generalized to other country or other sectors of activity. Therefore, it would be interesting to reproduce the study with managers of other country or activities sector in the public and private sectors. Fourth, the majority of participants were women (68%), it is then possible that there is a gender effect related to this sample. The results obtained are probably more representative of women and therefore not necessarily generalizable to both genders. Also, the fact that the set of measurement came from the same source (i.e., managers), leads to the possibility of a common variance bias. This study also used self-reported data. This type of data could include self-report biases (e.g., social desirability). Future research could incorporate a social desirability scale to better control for this aspect or consider including informant-reported measures of leadership form, for example, employees. Also, the internal consistency indices of certain leadership dimensions (e.g., *laissez-faire*, transactional) are lower than the desired value of 0.70, which indicates some measurement issues therefore caution should be exercised when interpreting the results of the study. But as reported by Dussault et al. (2013) in their validation study of the Leadership Self-Report Scale, the *laissez-faire* leadership had the lowest Cronbach’s alpha coefficient. Moreover, a recent study by Tóth-Király et al. (2023) using the measurement tool developed by Dussault et al. (2013) also obtained Cronbach’s alpha scores ranging from 0.465 to 0.671 for the *laissez-faire* leadership. As mentioned by Dussault et al. (2013), managers’ leadership behaviors associated with *laissez-faire* leadership are more difficult to assess because they are less valued than behaviors associated with transactional and transformational leadership. We thus encourage the research community to pursue efforts to validate French-language leadership scales that would be effective for different sectors of activity (e.g., healthcare). Finally, the specific contribution of organizational culture to managers’ psychological health will need to be evaluated in future studies against workplace and individual factors also contributing to well-being and psychological distress.

## Conclusion

While the consequences of leadership on employees’ psychological health are widely examined, few studies have focused on leadership antecedents as well as managers’ psychological health. In addition to looking at contextual antecedent of leadership, namely organizational culture, this paper examines managers’ psychological health as mediator. It also focuses on managers’ own perspective of their leadership which add another perspective to the literature. Our study provides a more in-depth understanding of the relationship between organizational culture and leadership styles. More specifically, our findings highlight the benefits of implementing a group organizational culture to enhance psychological well-being, reduce psychological distress symptoms and promote good leadership behaviors. Results also show the key role of managers’ psychological health as a resource to improve their capacity to adopt good leadership behaviors. Finally, this study highlights the need for further research to better understand the different conditions that promote managers’ psychological health and good leadership behaviors. Knowing these conditions will enable organizations to be better equipped to offer a healthy workplace that helps attract and retain employees and managers.

## Data availability statement

Access to the data is restricted to protect confidential information. The data could be available upon request with permission of the healthcare facility and participants. Requests to access these datasets should be directed to J-DG, julie.dextras-gauthier@fsa.ulaval.ca.

## Ethics statement

The studies involving human participants were reviewed and approved by Comité d’éthique de la recherche de l’Université Laval and the study protocol was approved by the three healthcare facility research ethics committees. The participants provided their written informed consent to participate in this study.

## Author contributions

JD-G and M-HG designed the study and both were responsible for the data collection, conducted the analyses, and interpreted the results. JD-G took the lead in the writing process. M-HG helped draft the manuscript. JD participated in the data collection and provided critical feedback for the manuscript. LA helped with the literature review and references. All authors contributed to the article and approved the submitted version.
